# 3,6-dihydroxyflavone suppresses the epithelial-mesenchymal transition in breast cancer cells by inhibiting the Notch signaling pathway

**DOI:** 10.1038/srep28858

**Published:** 2016-06-27

**Authors:** Junli Chen, Hui Chang, Xiaoli Peng, Yeyun Gu, Long Yi, Qianyong Zhang, Jundong Zhu, Mantian Mi

**Affiliations:** 1 Research Center for Nutrition and Food Safety, Third Military Medical University; Chongqing Key Laboratory of Nutrition and Food Safety, Chongqing, China; 2 Department of Public Health, School of Preclinical Medicine, Chengdu Medical College, Chengdu, China

## Abstract

The epithelial to mesenchymal transition (EMT) is a critical developmental program in cancer stem cell (CSC) maintenance and in cancer metastasis. Here, our study found that 3,6-DHF could effectively inhibit EMT in BC cells *in vitro* and *in vivo*. 3,6-DHF effectively inhibits the formation and proliferation of BCSCs, and consequently reduces the tumor-initiating capacity of tumor cells in NOD/SCID mice. Optical *in vivo* imaging of cancer metastasis showed that 3,6-DHF administration suppresses the lung metastasis of BC cells *in vivo*. Further studies indicated that 3,6-DHF down-regulates Notch1, NICD, Hes-1 and c-Myc, consequently decreasing the formation of the functional transcriptional unit of NICD-CSL-MAML, causing Notch signaling inactivation in BC cells. Over-expression of Notch1 or inhibition of miR-34a significantly reduced the inhibitory effects of 3,6-DHF on EMT, CSCs, as well as cells migration and invasion in BC cells. These data indicated that 3,6-DHF effectively inhibits EMT and CSCs, as well as cells migration and invasion in BC cells, in which miR-34a-mediated Notch1 down-regulation plays a crucial role.

Breast cancer (BC) is a common and leading cause of cancer deaths among women worldwide, particularly in Western countries^1^. BC damages have serious consequences for the health and quality of life for women. Despite advances in screening, diagnosis and therapy, BC continues to pose an enormous global healthcare problem^2^,^3^. The majority of breast cancer-related deaths are caused by highly metastatic tumors, in which the primary tumor cells migrate through blood capillaries or draining lymphatic vessels to new organ sites^4^. The epithelial to mesenchymal transition (EMT) is a critical developmental program in cancer progression, which is often activated during cancer invasion and metastasis^5^,^6^. During the EMT, epithelial cells are converted to migratory and invasive cells. This process is a fundamental event in morphogenesis because it is intimately involved in the generation of tissues and organs during the embryogenesis of both vertebrates and invertebrates. The EMT is a complex molecular program that primarily includes the loss of epithelial markers, such as E-cadherin, and the promotion of mesenchymal markers, such as snail, slug and twist^7^,^8^.

The EMT not only plays a key role in tumor metastasis, it is also associated with the acquisition of cancer stem cell (CSC) characteristics^9^,^10^. The inhibition of the EMT can suppress the formation of CSCs. Increasing evidence indicates that CSCs govern the enhanced tumorigenicity and capacity for proliferation, self-renewal and differentiation in various cancers^11^,^12^,^13^. In addition, CSCs are responsible for tumor initiation, progression, metastasis, drug-resistance and relapse^14^. Consequently, the inhibition of the EMT and the subsequent formation of CSCs is an attractive strategy for the treatment of cancer metastasis.

Notch signaling is an evolutionarily conserved pathway involved in cell fate control during development, stem cell self-renewal and postnatal tissue differentiation^15^,^16^. Accumulating evidence suggests that Notch signaling regulates many physiological processes, including cell fate determination during embryonic development, tissue maturity, and tumor cell proliferation, as well as CSC maintenance and the EMT^15^. Notch signaling is activated to release the intracellular domain of Notch (NICD), and the NICD cleavage products subsequently translocate to the nucleus and interact with a transcription complex to activate the Notch target genes Hes-1 and c-Myc^16^,^17^. Notch signaling is often aberrantly over-expressed in many cancers and increases the expression of Snail, Twist and Slug in epithelial cells to promote the EMT^15^,^18^,^19^.

We previously identified a promising anticancer agent, the flavonol compound 3,6-dihydroxyflavone (3,6-DHF), using pharmacodynamic experiments, and demonstrated that it is a potent natural chemopreventive agent against breast carcinogenesis *in vitro* and *in vivo*^20^. However, the effects of 3,6-DHF on the EMT and CSCs in BC cells have not been clearly defined. Notably, our previous study indicated that 3,6-DHF epigenetically up-regulates the expression of miR-34a^21^, which is a potent down-regulator of Notch1. Based on these data, we hypothesized that 3,6-DHF may down-regulate Notch1 expression via up-regulation of miR-34a in breast cancer cells and result in suppression of EMT. In the present study, we first assessed the effects of 3,6-DHF on the EMT, migration and invasion in BC cells and then investigated its effects on breast CSC (BCSC) formation and tumor-initiating capacity. Furthermore, we explored the role of the Notch1 signaling pathway in 3,6-DHF-induced inhibition of the EMT and BCSCs.

## Results

### 3,6-DHF inhibits the EMT in BC cells *in vitro* and *in vivo*

**Figure 1:**
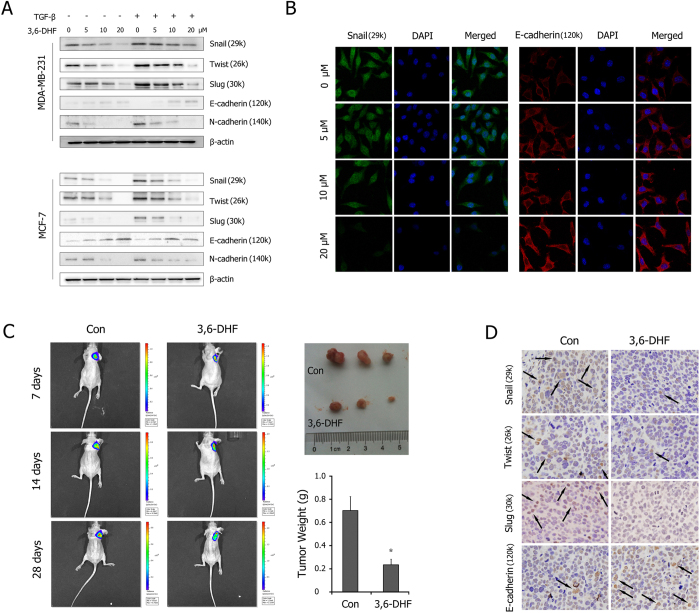
3,6-DHF inhibits EMT in BC cells *in vitro* and *in vivo.* (**A**) Western blot detections for the level of EMT markers in MDA-MB-231 and MCF-7 cells treated with different doses of 3,6-DHF (0, 5, 10, and 20 μM) for 24 h. (**B**) Immunofluorescence analysis for the level of snail and E-cadherin in MDA-MB-231 cells treated with different doses of 3,6-DHF for 24 h. (**C**) The effect of 3,6-DHF administration (20 mg/kg) on the growth of breast tumors monitored by *in vivo* luminescence imaging system. (**D**) Immunohistochemistry detections of snail, twist, slug and E-cadherin in breast tumors. The data are presented as the means ± SD (n = 3). Each data point represents the mean SEM of three independent experiments. ^*^P < 0.05 compared with control.

To evaluate the effects of 3,6-DHF on EMT, we first assessed the changes in the expression of EMT markers in BC cells using western blots. As shown in Fig. 1A, 3,6-DHF treatment resulted in significantly decreased levels of the mesenchymal markers snail, twist, slug and N-cadherin in a dose-dependent manner in MDA-MB-231 cells and MCF-7 cells. The results also showed that the levels of the epithelial marker E-cadherin increased in 3,6-DHF-treated BC cells. We promoted the EMT in BC cells by adding 10 ng/ml of transforming growth factor-β (TGF-β). The results indicated that TGF-β induced the over-expression of snail, twist, slug and N-cadherin and the down-regulation of E-cadherin, which was effectively mitigated with 3,6-DHF co-treatment. Accordingly, immunofluorescence analysis confirmed that 3,6-DHF induced the down-regulation of snail and up-regulation of E-cadherin in BC cells in a dose-dependent manner (Fig. 1B).

We further assessed the inhibitory effects of 3,6-DHF on the EMT in BC cells *in vivo*. A luciferase-expressing breast cancer cell line, MDA-MB-231-Luc-GFP, was injected into the mammary fat pad of female BALB/c mice. Stable expression of firefly luciferase and an *in vivo* luminescence imaging system (IVIS) allows for the longitudinal monitoring of tumor growth and metastasis. At day 7 post-implantation, the animals were imaged for luciferase activity, and all mice with similar tumor loads were randomized and separated into two treatment groups. After an additional 3 weeks of treatment, the tumors were isolated from the mice and immunohistochemistry detections were performed. As shown in Fig. 1C, 3,6-DHF administration (20 mg/kg) reduced the intensity and size of the *in vivo* luminescence in the animals, effectively inhibiting the growth of tumors. Immunohistochemistry detection (Fig. 1D) also showed that 3,6-DHF administration significantly suppressed the expression of snail, twist and slug and increased the level of E-cadherin in BC cells *in vivo*.

### 3,6-DHF inhibits BCSCs *in vitro* and *in vivo*

**Figure 2:**
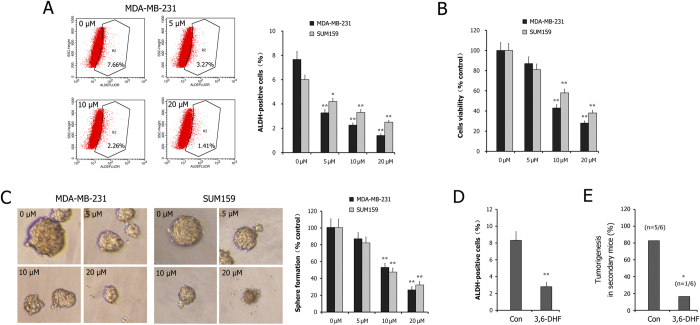
3,6-DHF inhibits BCSCs *in vitro* and *in vivo.* (**A**) 3,6-DHF treatment significantly decreased the percentage of ALDH-positive population in BC cells MDA-MB-231 and SUM-159. (**B**) Effects of 3,6-DHF treatment on cells viability in BCSCs detected by CCK-8 assay. (**C**) Mammosphere formation assay of non-adherent spherical clusters in BC cells treated with different dose of 3,6-DHF. (**D**) 3,6-DHF administration (20 mg/kg) significantly reduced the ALDH-positive populations in tumor cells in athymic mice. (**E**) The tumor-initiating capacity of the tumor cells derived from primary xenografts in NOD/SCID mice. The data are presented as the means ± SD (n = 3). Each data point represents the mean SEM of three independent experiments. ^*^P < 0.05, ^**^P < 0.01 compared with control.

We evaluated the effects of 3,6-DHF on BCSC formation using the ALDEFLUOR assay for aldehyde dehydrogenase (ALDH) activity. The results (Fig. 2A) showed that the 3,6-DHF treatment significantly decreased the percentage of the ALDH-positive population of BC cells. A mammosphere formation assay showed that the number of non-adherent spherical clusters decreased and that the size of the spheres were significantly reduced in 3,6-DHF-treated BC cells (Fig. 2C). CCK-8 assay showed that 3,6-DHF effectively reduces the cells viability of BCSCs and inhibits cell proliferation (Fig. 2B).

Then, we tested the inhibitory effects of 3,6-DHF on BCSCs *in vivo*. Breast tumors were isolated from mice and the tumor cells were analyzed. The results showed that 3,6-DHF administration significantly reduced the ALDH-positive population in tumor cells compared with the control mice (Fig. 2D). Moreover, we examined the tumor-initiating capacity of tumor cells in NOD/SCID mice. After inoculation for 30 d, the 6 inoculations of tumor cells from control xenografts initiated 5 tumors, whereas the tumor cells derived from 3,6-DHF-administered mice resulted in only 1 tumor (Fig. 2E). These data indicate that 3,6-DHF inhibits the formation of BCSCs *in vitro* and *in vivo*.

### 3,6-DHF inhibits migration and invasion of BC cells *in vitro* and *in vivo*

**Figure 3:**
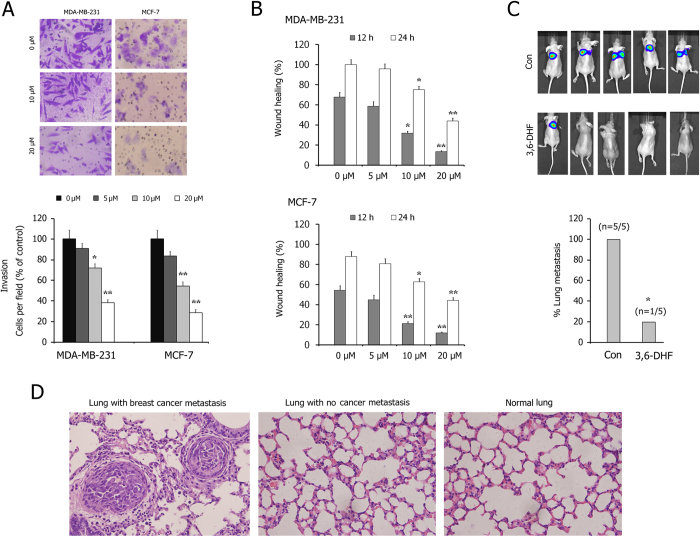
3,6-DHF inhibits migration and invasion of BC cells *in vitro* and *in vivo.* (**A**) Invasion assays in 3,6-DHF-treated BC cells using Transwell chambers. (**B**) The wound-healing assays in 3,6-DHF-treated BC cells. (**C**) The effect of 3,6-DHF on BC metastasis *in vivo* by a lung metastasis model. MDA-MB-231-Luc-GFP cells were injected via the lateral tail veins and optical *in vivo* imaging of cancer metastasis was monitored with IVIS. (**D**) Representative images of corresponding hematoxylin-eosin-stained lung sections at 5 weeks post-injection. The data are presented as the means ± SD (n = 3). Each data point represents the mean SEM of three independent experiments. ^*^P < 0.05, ^**^P < 0.01 compared with control.

Transwell assays showed that 3,6-DHF treatment effectively inhibited the invasion of BC cells through the Matrigel platform in a dose-dependent manner (Fig. 3A). Similarly, the wound-healing assay also showed that BC cells have high proliferation and mobility for wound healing, which is effectively suppressed by 3,6-DHF treatment (Fig. 3B).

Furthermore, we investigated the effect of 3,6-DHF on BC metastasis *in vivo* using a lung metastasis model. After a 1-week administration of 3,6-DHF, the mice were injected with MDA-MB-231-Luc-GFP via the lateral tail veins. Optical *in vivo* imaging of cancer metastasis was monitored with IVIS. As shown in Fig. 3C, bioluminescent images showed lung metastasis at 5 weeks post-injection. All 5 control nude mice appeared lung metastasis, whereas 3,6-DHF-administered mice resulted in only 1 lung metastasis. HE staining (Fig. 3D) confirmed that the lung nodules in these mice were BC metastasis. 3,6-DHF administration significantly inhibited the lung metastasis of BC cells *in vivo*.

### 3,6-DHF suppresses Notch signaling pathway in BC cells

**Figure 4:**
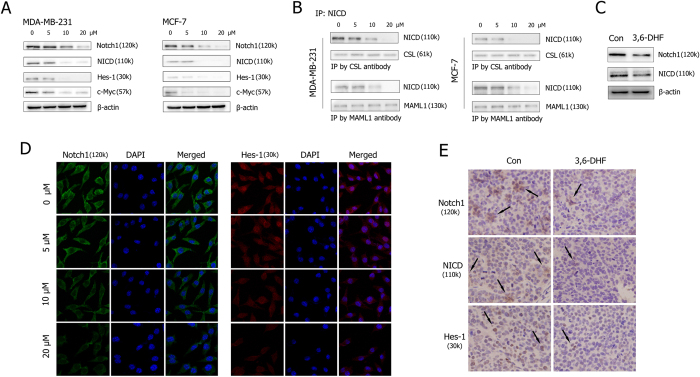
3,6-DHF suppresses Notch signaling pathway in BC cells. (**A**) Western blot detections for the level of Notch1, NICD, Hes-1 and c-Myc in MDA-MB-231 and MCF-7 cells treated with different doses of 3,6-DHF (0, 5, 10, and 20 μM) for 24 h. (**B**) The effect of 3,6-DHF treatment for 24 h on NICD-CSL-MAML functional unit formation in MDA-MB-231 cells detected by immunoprecipitation combined western blots. (**C**) Western blot detections for the level of Notch1 and NICD in breast tumors. (**D**) Immunofluorescence detections of Notch1 and Hes-1 levels in MDA-MB-231 cells treated with different doses of 3,6-DHF (0, 5, 10, and 20 μM) for 24 h. (**E**) Immunohistochemistry detections of Notch1, NICD and Hes-1 expression in breast tumors.

As shown in Fig. 4A, 3,6-DHF treatment decreased the levels of Notch1, NICD, Hes-1 and c-Myc in a dose-dependent manner. Immunofluorescence and immunohistochemistry assays confirmed the 3,6-DHF-induced down-regulation of Notch1, NICD and Hes-1 *in vitro* and *in vivo* (Fig. 4D,E). Western blots detections also showed that 3,6-DHF administration significantly decreased the expression of Notch1 and NICD in xenograft breast tumors (Fig. 4C). We then performed immunoprecipitation combined with western blots to determine the functional unit of formation. The results (Fig. 4B) showed that significantly less NICD was isolated with the CSL and MAML1 antibodies in 3,6-DHF-treated BC cells, indicating that 3,6-DHF caused Notch signaling inactivation.

### Notch1 over-expression reduces the inhibitory effects of 3,6-DHF on the EMT and BCSCs

**Figure 5:**
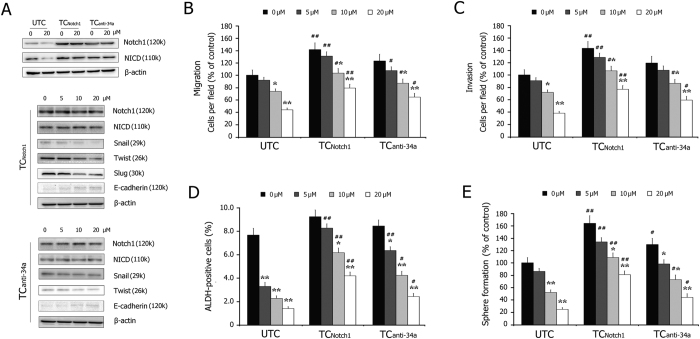
Notch1 over-expression reduces the inhibitory effects of 3,6-DHF on EMT and BCSCs. (**A**) Effects of 3,6-DHF treatment for 24 h on the expressions of Notch1, NICD, snail, twist, slug and E-cadherin in un-transfected MDA-MB-231 cells (UTC), cells transfected with the plasmid of pcDNA3-Notch1 (TC_Notch1_) and cells transfected with miR-34a inhibitor (TC_anti-34a_). Effects of 3,6-DHF treatment for 24 h on the migration (**B**), invasion (**C**), ALDH-positive population (**D**) and mammosphere formation (**E**) in UTC, TC_Notch1_ and TC_anti-34a_ cells. The data are presented as the means ± SD (n = 3). Each data point represents the mean SEM of three independent experiments. ^*^P < 0.05, ^**^P < 0.01 compared with control; ^**#**^*P* < 0.05, ^**##**^*P* < 0.01 compared with UTC.

To explore the role of Notch signaling in the 3,6-DHF-induced inhibition of EMT and BCSCs, we transfected MDA-MB-231 cells with either the plasmid pcDNA3.1-Notch1 (TC_Notch1_) or a locked nucleic acid oligonucleotide complementary to the miR-34a sequence (TC_anti-34a_). As shown in Fig. 5A, the transfection of pcDNA3.1-Notch1 plasmids or the miR-34a inhibitor led to significant production of Notch1 in MDA-MB-231 cells and blocked the effect of 3,6-DHF on Notch1 expression. The over-expression of Notch1 reduced the suppressive effects of 3,6-DHF on the EMT markers snail, twist and slug and weakened the up-regulatory effect of 3,6-DHF on E-cadherin. Further studies showed that Notch1 over-expression significantly compromised the inhibitory effects of 3,6-DHF on BC cell migration and invasion (Fig. 5B,C), as well as the percentage of the ALDH-positive population and mammosphere formations (Fig. 5D,E). These data indicate that miR-34a-mediated Notch1 down-regulation plays a crucial role in the 3,6-DHF-induced inhibitory effects on the EMT and BCSCs, as well as migration and invasion.

## Discussion

Cancer metastasis is the primary cause of most cancer death, rather than primary tumors^19^,^22^. The EMT is a cellular process in which epithelial cells lose their cell polarity and cell-cell adhesion and gain migratory and invasive properties to become mesenchymal cells^23^. During the EMT, epithelial cells lose their junctions and apical-basal polarity, reorganize their cytoskeleton, undergo a change in the signaling programs that define cell shape and reprogram gene expression. This increases the motility of individual cells and enables the development of an invasive phenotype^24^. The EMT is integral to development, and the processes underlying it are reactivated in wound healing, fibrosis and cancer progression^25^. The EMT plays important roles in the progression and metastasis of cancer and is a critical step of cancer cell evolution and CSC formation. The CSC hypothesis proposes that cancers are driven by a small subpopulation of stem-like cells, which possess such capacities as self-renewal, differentiation, tumorigenesis, and resistance to radiation therapy and chemotherapy^26^. Clinical analyses of CSCs in breast tumors found a correlation between the proportion of CSCs and a poor prognosis. The concept of CSCs has profound clinical implications for cancer prevention and therapeutic strategies. Chemoprevention and therapeutic strategies that specifically target CSCs are urgently needed^27^. Taken together, the inhibition of the EMT and CSCs is a key focus for cancer prevention and control. Dietary flavonoids, a large group of polyphenolic compounds in fruits and vegetables, have been identified as potential anti-cancer components in the diet. The present study demonstrates that the flavonol 3,6-DHF effectively inhibits the EMT in BC cells and eliminates BCSCs *in vitro* and *in vivo*. Consequently, 3,6-DHF reduces migration, invasion and the tumor-initiating capacity of BC cells. These findings provide new knowledge and useful insight into the anti-cancer effects and mechanisms of dietary flavonoids.

The Notch signaling pathway is associated with the regulation of cell fate during several distinct developmental stages of the mammary gland and is related to the development and maintenance of CSCs, possibly via the initiation of EMT-like processes^28^,^29^,^30^. The aberrant activation of Notch signaling is associated with the development and progression of several human malignancies, including BC. After Notch activation, NICD translocates to the nucleus and binds to CSL, a constitutive transcriptional repressor, displacing corepressors and recruiting coactivators, such as MAML proteins^31^. In EMT processes, Notch cross talks with several transcription and growth factors relevant to the EMT, including Snail, Slug, TGF-β, FGF, and PDGF^32^. Our study showed that 3,6-DHF inhibits the expression of Notch1 and consequently suppresses the Notch signaling pathway in BC cells. Further studies indicated that Notch1 over-expression reduces the inhibitory effects of 3,6-DHF on the EMT and BCSCs. These data suggest that 3,6-DHF inhibits the EMT and BCSCs by inhibiting the Notch signaling pathway.

miRNAs play important roles in the pathogenesis of human diseases, including malignancy, and may function as both oncogenes and tumor suppressors^33^. miR-34a is a potent tumor suppressor, a direct transcriptional target of p53, and a component of the p53 transcriptional network^34^,^35^. Moreover, Notch1 is a direct target of miR-34a, and miR-34a negatively regulates the Notch signaling pathway^36^,^37^,^38^. Our previous study found that 3,6-DHF epigenetically up-regulates the expression of miR-34a in BC cells. The present study further revealed that 3,6-DHF inhibits Notch1 expression via the up-regulation of miR-34a and suppresses Notch signaling, resulting in the inhibition of EMT and BCSCs. Nevertheless, it’s worthy to note that after the blockage of 3,6-DHF–induced down-regulation of Notch1, the compound still exerts effects in part on EMT, though the effects were decreased significantly. So it indicated that 3,6-DHF could suppress EMT via some extra-Notch pathway. The detailed mechanisms need further exploring and investigating in the future. In all, our findings highly suggest that 3,6-DHF may be an effective therapeutic strategy for metastatic breast cancer without side effects.

## Materials and Methods

### Ethics statement

Female NOD/SCID mice and female BALB/c nude mice, 6–8 week old, weighing 20–25 g were used. All of the animal procedures involving mice, such as housing and care, and experimental protocols were approved by Institutional Animal Care and Use Committee (IACUC) of the Third Military Medical University (Chongqing, China) (approval SYXC-2014-00012). All procedures performed on the rats were conducted according to the guidelines from the National Institutes of Health. All surgeries were performed under chloral hydrate (5%) anesthesia, and all efforts were made to minimize suffering to the animals.

### Cell lines and culture

Human breast cancer cell lines were purchased from Institute of Biochemistry and Cell Biology, Chinese Academy of Sciences (Shanghai, China). All cell lines have been tested and authenticated by DNA (short tandem repeat (STR) genotyping) profiling. Isolated BCSCs were plated in serum-free DMEM supplemented with 1% BSA, 5 μg/ml insulin, 10 ng/ml bFGF, 20 ng/ml EGF, 1 μg/ml hydrocortisone and B-27 in a low cell-binding dish as previously described^39^. After mammospheres formed, BCSCs were trypsinized and evaluated for stem cell markers by flow cytometry and then cultured in DMEM containing 10% FBS for 24 h before treatment with 3,6-DHF or other reagents. The cells were maintained at 37 °C in a humidified incubator in an atmosphere containing 5% CO_2_.

### Mammosphere formation assay

Single-cell suspensions were prepared and cultured at a density of 1000–2000 cells/ml in six-well ultralow attachment plates (Corning, NY, USA). A mammosphere assay was also performed in DMEM/F-12 media containing 10 μg/ml insulin, 0.5 mg/ml hydrocortisone, 2% B27, 20 ng/ml epidermal growth factor, 10 ng/ml bFGF and 0.4% bovine serum albumin. After 7 days of incubation, the number of mammospheres was counted under a microscope and the photos were acquired.

### Migration and invasion assays

The migration and invasion assays were performed and evaluated using a Transwell chamber (Corning, NY, USA) as described previously^40^. For migration assays, 2 × 10^4^ cells in serum-free media were placed into the upper chamber. For invasion assays, 1 × 10^5^ cells in serum-free media were placed into the upper chamber with an insert coated with Matrigel (BD Biosciences, San Jose, CA, USA).

### Wound healing assay

Cells were cultured in six-well plates until they reached 100% confluence. A vertical or horizontal wound was gently created in monolayers using a 20 μl sterile pipette tip. The cells were then washed for 3 times with growth medium to remove the detached cells, and the medium was added with fresh medium treated with 3,6-DHF. Images were captured using an inverted microscope and camera at designed times to assess the inhibition of wound closure.

### ALDEFLUOR assay

ALDEFLUOR assay was performed using an ALDEFLUOR assay kit according to the manufacturer’s recommendations. Green fluorescence positive cells in live cells were analyzed by flow cytometry (FCM, Beckman Coulter, USA) by comparing the fluorescence intensity of the DEAB treated sample; these cells will be represented as cells with high ALDH activity (ALDH^+^ cells).

### Western blot analysis

All antibody assays were conducted following the western blot manufacturer’s protocol. The total cell lysates were prepared using RIPA buffer (25 mM Tris-HCl, pH 7.6, 150 mM NaCl, 1% NP40, 1% sodium deoxycholate, and 0.1% sodium dodecyl sulfate (SDS)) supplemented with protease and phosphatase inhibitors. Equal amounts of cellular proteins were resolved by electrophoresis in 10% or 12% SDS-polyacrylamide gels for western immunoblotting with specific antibodies. The antigen-antibody complexes on the filters were detected by chemiluminescence. Finally, the blots were scanned, and densitometric analysis was performed on the scanned images using Scion Image-Release Beta 4.02 software.

### Immunofluorescence

The cells were fixed with 4% paraformaldehyde and permeabilized in 1% Triton 100 for 15 min. The slides were blocked with 2% donkey serum for 0.5 h and incubated with antibodies at 4 °C overnight. The slides were rinsed and incubated with secondary antibodies at 37 °C for 2 h. The nuclei were counterstained with DAPI (1: 1000), and the slides were immediately analyzed via laser confocal scanning microscopy.

### Immunohistochemistry

The tissue sections (4-μm-thick) were placed onto treated slides, heat-fixed, deparaffinized, rehydrated, and subjected to antigen retrieval for immunohistochemistry, Sections were stained for H&E for morphological study. After washing with PBS, the slides were blocked with 2% serum for 0.5 h and then incubated with the primary antibodies at 4 °C overnight. The secondary biotinylated antibody was then applied, and the signal was developed using a modified avidin-biotin complex immunoperoxidase staining procedure. Counterstaining was performed with Trypan blue or Harris hematoxylin.

### Coimmunoprecipitation

Treated cells were washed in cold PBS, then harvested ice-cold lysis buffer (40 mM HEPES, pH 7.4, 2 mM EDTA, 10 mM β-glycerophosphate, 0.3% (w/v) CHAPS, and protease inhibitors). The soluble fractions of the cell lysates were isolated by centrifugation at 14,000 g for 15 min. The lysate was precleared by incubation with 20 μl of protein A-Agarose and 1 μg of control rabbit IgG for 1.5 h. For co-immunoprecipitation, an antibody CSL or MAML was added to the lysates, and the lysates were incubated with rotation for 2 h at 4 °C. 20% Protein A-sepharose was then added, and the lysates were incubated for an additional 1 h at 4 °C. The lysate was centrifuged at 14,000 g for 5 s. The immunoprecipitates were washed three times with PBS. The immunoprecipitated proteins were denatured by the addition of 20 ml of sample buffer and boiling for 5 min, resolved by 8–16% SDS-PAGE, and analysed by western blot.

### Plasmids, oligonucleotides and transfections

Anti-miR-34a oligonucleotides (5′-ACAACCAGCTAAGACACTGCC-3′) were obtained from Exiqon (Denmark). The pcDNA3.1-Notch1 plasmid was purchased from GeneChem (shanghai, China). The cells were transfected using Lipofectamine 2000 in Opti-Mem according to the manufacturer’s protocol. The medium was replaced 6 h later, and the cells were collected for the subsequent experiments 48 h post-transfection. The final concentrations of oligonucleotides were 100 nM.

### qRT-PCR analysis

The total RNA was extracted using Biozol reagent. The miRNA first-strand cDNA synthesis kit and miRNA Real-Time PCR Assay kit (aidlab, Beijing) were used to quantify the miRNA transcripts in our study following the manufacturer’s instructions. Each reaction sample was run in triplicate. The expression of U6 (F-CTCGCTTCGGCAGCACA, R-AACGCTTCACGAATTTGCGT) small nucleolar RNA was used as a control. All experiments were carried out in triplicate. The data were analyzed according to the comparative CT method (2^−ΔΔCt^).

### Xenografted MDA-MB-231 cells in athymic mice

Female BALB/c nude mice were randomly divided into the control and 3,6-DHF administration groups. These rats were then implanted with Luc-MDA-MB-231 cells at a density of 5 × 10^6^ cells/0.1 ml PBS s.c. into the right axilla. Seven days after implantation, the mice were i.g. orally fed 3,6-DHF (20 mg/kg/day) or vehicle alone (normal saline). During the experiments, the rats were weighed twice per week and the tumors were measured once every four days during the treatment period of 28 days. Mice under anaesthesia were injected intraperitoneally with 15 mg/ml of D-luciferin (Sinochrome, shanghai, China) in DPBS, and images were recorded by the IVIS Imaging System (PerkinElmer, USA) after the injection. Observations by IVIS were continued once a week, immediately after the injection, up to 4 weeks. Tumors were measured with a caliper and the volume was calculated using V = (width^2^ × length)/2. Mice were sacrificed and examined.

### Lung metastasis model

Female BALB/c nude mice were randomly divided into the control and 3,6-DHF (20 mg/kg/day) administration groups. Cancer cell suspensions were transplanted into the tail veins of athymic nude mice (BALB/c; 1 × 10^6^ cells per mouse) for a mouse model of pulmonary metastasis after intravenous transplantation. After 4 or 5 weeks, optical *in vivo* imaging of cancer metastasis was monitored with IVIS. Bioluminescent images were acquired 5 min after the intraperitoneal injection of the D-luciferin solution (Sinochrome, shanghai, China). Mice were anaesthetized with 2.5% isoflurane gas in the oxygen flow (1.5 ml/min) during imaging. The signal intensity was quantified and analysed using Living Image 2.50-IgorPro4.90 software.

### Secondary NOD/SCID mouse model

Xenografted MDA-MB-231 cells in athymic mice were treated as mentioned above. Xenografts from 3,6-DHF-administrated mice and control were isolated and living cells were sorted out from the dissociated tumors. Each NOD/SCID mouse was inoculated with 1 × 10^6^ cells from control mouse tumors in one side of inguinal fat pad and another 1 × 10^6^ cells from 3,6-DHF-treated tumors in the contralateral fat pad.

### Statistical analysis

The results are presented as the means ± standard deviation (SD) from at least three independent experiments. The tumour incidences were compared using the χ2 test. The other data were analysed by one-way ANOVA followed by Tukey’s test for multiple comparisons. The difference significance was set at P < 0.05.

## Additional Information

**How to cite this article**: Chen, J. *et al*. 3,6-dihydroxyflavone suppresses the epithelial-mesenchymal transition in breast cancer cells by inhibiting the Notch signaling pathway. *Sci. Rep.* **6**, 28858; doi: 10.1038/srep28858 (2016).
